# Towards digital healthy ageing: the case of Agatha and priorities moving forward

**DOI:** 10.1016/j.lanwpc.2022.100649

**Published:** 2023-02-06

**Authors:** Maria Isabella Gariboldi, Mengji Chen, Yuxin Wei, Shan Xu, Gauden Galea, Siwon Lee

**Affiliations:** a Healthy Ageing Unit, Division of Healthy Environments and Populations, World Health Organization (WHO), Western Pacific Regional Office (WPRO), 669 Ermita Manila 1000, Metro Manila, Philippines; b Innovation and Research Unit, Division of Data, Strategy and Innovation, World Health Organization (WHO), Western Pacific Regional Office (WPRO), 669 Ermita Manila 1000, Metro Manila, Philippines; c WHO Collaborating Centre for Digital Health China Academy of Information and Communications Technology (CAICT), 52 Hua Yuan Bei Road, Haidian District Beijing, 100191, PR China; d Shanghai Institute of Major Infectious Disease and Biosafety, Fudan University, 220 Handan Road. Shanghai, 200433, China; e WHO Representative Office in China, 401 Dongwai Diplomatic Building 23, Dongzhimenwai Dajie Chaoyang District Beijing, 100600, PR China

**Keywords:** Healthy ageing, Digital health, Older people, Healthy longevity, Social determinants of health, Older adults, Population ageing, Innovation, Health promotion

## Abstract

Digital tools have an important role to play in meeting the health demands of ageing societies. However, current technological design paradigms often marginalize older people. We adopted a lean, user-centred approach to prototype the Avatar for Global Access to Technology for Healthy Ageing (Agatha), an interactive one-stop shop for healthy ageing promotion. Building on this experience, we present a vision for an integrated approach to “digital healthy ageing”. Older people consulted predominantly associated “healthy ageing” with disease avoidance. Digital healthy ageing should take a more holistic approach, covering self-care, prevention, and active ageing. It should also consider social determinants of health in old age, including access to information and digital health literacy, as they interact with poverty, education, access to health services and other structural factors. We use this framework to map out key areas of innovation and explore policy priorities and opportunities for innovation practitioners.

## Introduction

Decreasing fertility rates and increasing life expectancy are contributing to global population ageing. As of 2019, there were 703 million people above the age of 65, corresponding to 9% of the global population, this number being projected to reach 1.5 billion – or one in six people – by 2050.^1^ Promoting healthy ageing, defined as “the process of developing and maintaining the functional ability that enables wellbeing in older age”,^2^ is both crucial to reducing the healthspan-lifespan gap, and to supporting the diverse contributions older people can make to society. The Regional Action Plan on Healthy Ageing in the Western Pacific highlights how ageing requires both a health systems and a societal transformation with a key facilitating role to be played by technological innovation.^3^ Data-enabled technologies – such as smartphone apps, artificial intelligence (AI), wearables, robotics and the internet of things (IoT) – are extending their impacts by facilitating the generation, interpretation and communication of information. These digital tools can directly support health by nudging health behaviour change, tracking gradual changes in health, facilitating interactions with the health system, and providing health information.

Despite the benefits of digital tools and the growing “silver economy”, older people remain largely digitally marginalized, due to a number of barriers including low digital literacy, reduced confidence and motivation to use technologies, as well as ageist product designs and marketing. This is exacerbated by the gross underrepresentation of older people among technology developers.^4^ In light of these barriers, participatory and human-centred design approaches are fundamental to bridging the technology usability and desirability gap for older people. The Innovation and Research and the Healthy Ageing Units at the Western Pacific Regional Office (WPRO) of the World Health Organization (WHO) have set out to prototype the Avatar for Global Access to Technology for Healthy Ageing (Agatha). The purpose of Agatha is to both serve as a one-stop shop platform for healthy ageing promotion, as well as to encourage inclusive design principles for digital tools for older people. In the first part of this article, we make a case for applying human-centred, agile methodologies for the development of digital health tools for older people using Agatha as a case study. In the second part of this article, we present our vision for transitioning from digital health for older people to *digital healthy ageing*. This vision provides a more holistic framework for mapping high-impact areas in which inclusive design of digital technologies can benefit older people's health rooted in a social determinants of health approach to maintaining intrinsic capacity and functional ability. Finally, we synthesize priorities and opportunities for the public and private sectors in working towards this vision.

## The development of Agatha

Agatha was originally envisioned as an avatar chatbot that could answer questions about COVID-19 and healthy ageing based on WHO guidelines. An avatar interface was chosen with the specific goal of improving the accessibility of health information for older people. Namely, voice activation was assumed to overcome commonly reported barriers for older people such as difficult-to-read text and the need to type or push small buttons.^5^ Integration in one platform and a simple interface were chosen to improve usability. Finally, a humanoid appearance was thought to enable a more natural, human-like interaction. Agatha was also designed to be an older person, with the intention of promoting social inclusion by representing older people in active roles. The first prototype of Agatha was developed by the WHO Collaborating Centre for Digital Health – the China Academy of Information and Communications Technology (CAICT) – in June 2021.

**Fig. 1:**
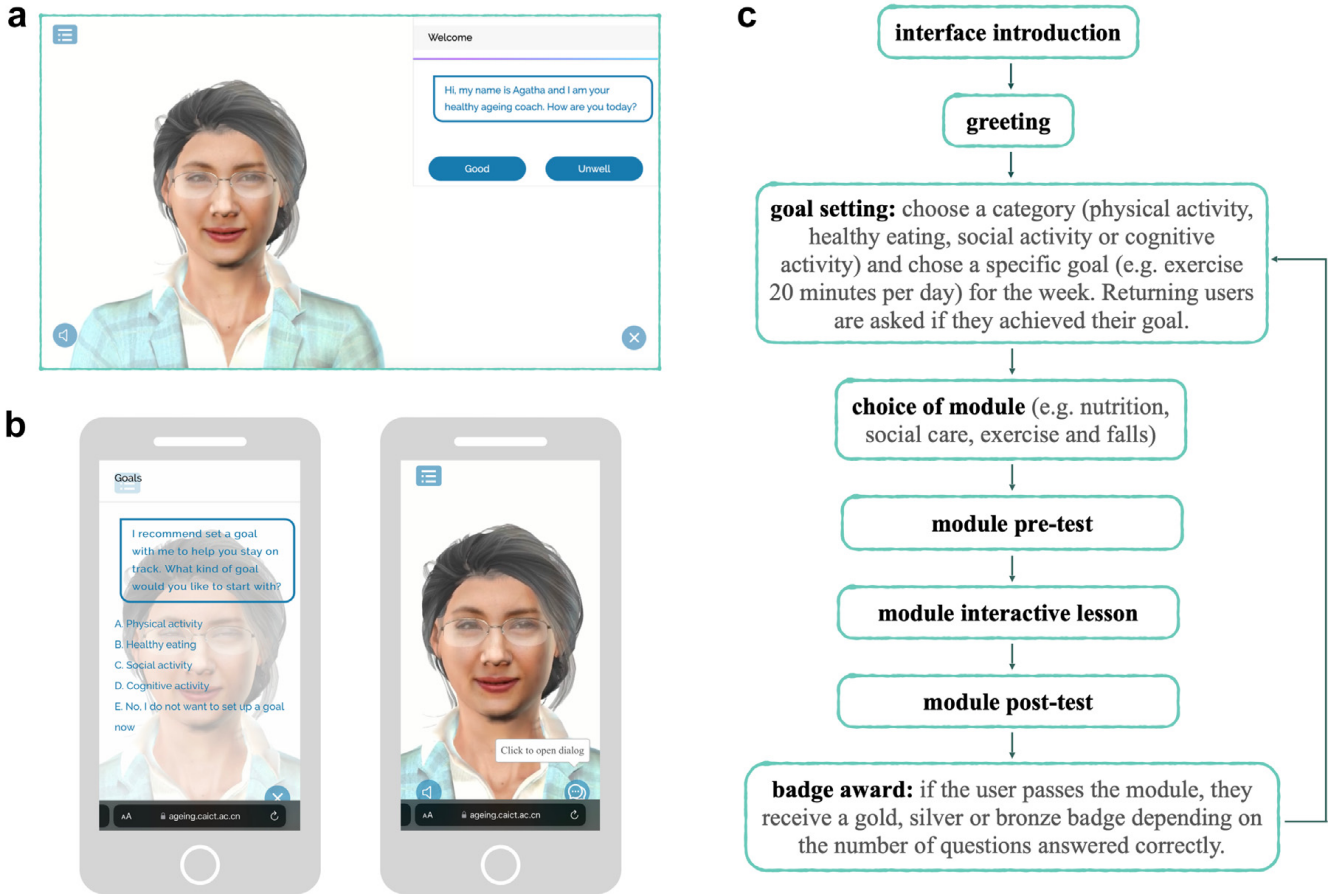
**High-level overview of the Agatha prototype**. (a) Web application interface, (b) H5 mobile application interface and (c) a simplified representation of the user journey.

A lean approach, consisting of iterative development stages integrating frequent user feedback, was adopted. Research on older people as users of digital tools as well as consumers of health information was conducted alongside more targeted user testing using a combination of methods including observation and focus group interviews. This was carried out in the Philippines by the Institute on Aging, University of the Philippines Manila and in China by Pinetree Care Group. Throughout the process, based on user feedback, Agatha was repositioned from being an interactive but passive digital health worker, in the form of an avatar chatbot answering older people's questions, to a healthy ageing coach directing older people's user journey through selected content in the form of interactive learning modules including quizzes and gamification elements. Fig. 1 shows Agatha's current interface as well as the platform's high-level interaction flow. It must be noted that Agatha is intended as a proof-of-concept prototype, not a final product. Her design is still in the process of being improved and her content is currently limited to WHO guidance on selected health topics as well as the Integrated Care for Older People (ICOPE), a person-centred guidance for mitigating declines in intrinsic capacity with a focus on self-management as well as social care and caregiver support.^6^ ICOPE guidance encompasses six domains of intrinsic capacity: cognitive decline, limited mobility, visual impairment, malnutrition, hearing loss and depressive symptoms. Three of these domains are currently integrated into Agatha's content through modules on exercise and falls, social care, and nutrition.

The Agatha prototype is currently available in both English and Mandarin, as a web application accessible through computers and as an H5 mobile application, a browser-based application optimized for user experience and easily shareable through WeChat. Future upgrades to Agatha will likely include the development of a smartphone application. Possible content expansions include linking to selected external resources, improvements to dialogue flexibility, and the use of nudges in the form of notifications. Furthermore, the possibility of using Agatha for older people to conduct self-assessments through an interactive interface is being considered. Upcoming user testing will focus on longer-term user behaviour through a pilot in Malaysia and possible localization through small-scale integration with community health clinics.

## Reflections from the development of digital tools for healthy ageing

### From digital health assistant to coach: shaping conceptions of healthy ageing

One of the findings uncovered in the early phases of testing was the disease-oriented nature of questions posed by users to Agatha. At this initial stage of testing, Agatha consisted in an interactive chatbot to answer older people's questions. Knowing Agatha's content was limited to ICOPE and COVID-19 guidance, users nonetheless expressed the need to obtain information related to specific diseases. This posed a technical limitation given the breadth a corpus would require to address all relevant disease areas and the inherent potential risks of providing advice on medical issues. Moreover, this tendency highlighted a clear mismatch between designers' and users' conceptions of healthy ageing.

Older people's expectation of the content is reminiscent of Nakatani's representation of the emerging model of health with population ageing.^7^ Health has been conventionally understood as the absence of disease. This view is reinforced by the classic conceptualization of disease occurrence as a binary switch triggered by individual episodes. While this approach may be an appropriate conceptualization of infectious diseases, the emerging paradigm that considers transitions from health to disease as gradual processes driven by a variety of factors – many of which beyond the traditional domain of health systems – better embodies the progression of non-communicable diseases. This paradigm shift is particularly important in the context of healthy ageing, which requires addressing the gradual decline in intrinsic capacity and in which self-care, preventative and proactive behaviours, as well as social and environmental contributors, take a central role.^3^^,^^6^

This early finding is a powerful reminder that promoting healthy ageing requires both a health systems and a societal transformation.^3^ It serves as a valuable lesson that digital healthy ageing requires holistic and integrated approaches beyond the conventional domains of digital health. Recognizing the mismatch between our value proposition for Agatha and intended users’ expectation of a virtual health assistant, Agatha was repositioned as a healthy ageing coach to promote healthy ageing as a gradual process, instead of following user needs under the dichotomy of health and disease. The fundamental difference between an assistant and a coach is that a coach sets the agenda whereas an assistant follows instructions. As a coach, Agatha can actively promote healthy ageing information through pre-designed interaction flows aimed at educating older people on concrete behaviours they can adopt to positively influence their health. This repositioning also enabled the incorporation of promising behavioural science approaches in the form of gamification.^8^

### Combining human-centred and lean start-up approaches for better solutions

The lean start-up approach and human-centred design are methodologies for product development that have been widely applied in the private sector. Eisenmann et al. (2013) describe the lean start-up approach as a form of hypothesis-driven entrepreneurship whose goal is to maximize information generation and minimize resource utilization to resolve uncertainty. This is done by generating “falsifiable business model hypotheses” tested iteratively through the use of minimum viable products (MVP).^9^ Human-centred design, on the other hand, is a design approach that puts target users’ needs at the centre and places a strong emphasis on empathy as a driver of creativity and innovation.^10^ Despite the growing significance of older users as a demographic, few ventures set out to specifically target their needs. As a poorly served and poorly understood demographic, iterative design and continuous feedback processes aimed at engaging, understanding and empathizing with users are particularly important. Furthermore, older people have more complex user needs, given they are often subject to limited access to technology, are underrepresented in the workforce and are frequent targets of stereotyping. In the development of Agatha, users were engaged from the early stages to both obtain feedback as well as to more broadly understand their preferences and behaviours in the context of technology use and health information seeking. This permitted the platform to be repositioned early on resulting in significant improvements in user feedback.

Finally, engaging users enabled us to reframe our perspective on digital health innovations from one focused on implementing new tools towards one focused on service design with digital tools serving as enablers. Digital health innovation should not start with an interest in exploring the use of advanced technologies but by understanding user needs, exploring health visions, designing a value proposition and utilizing technology as a catalyst.

## Building towards digital healthy ageing: priorities and opportunities ahead

### Sketching a vision

Social determinants of health provide a useful lens to address the underlying drivers of health. Socioeconomic factors and physical environments, for example, affect both our access to health-enabling resources and our awareness and ability to engage in healthy behaviours.^11^ Several initiatives have set out to support healthy ageing by addressing the social determinants of health. The Seoul 50+ Foundation (https://50plus.or.kr/org/eng.do), for example, provides several resources such as training and life planning services in areas including work, finance, social integration and leisure. The University of the Third Age (U3A), started in France in 1972, is a model that promotes lifelong learning and knowledge sharing and has been successfully adapted in several countries to provide education and learning opportunities for older people.^12^

**Fig. 2:**
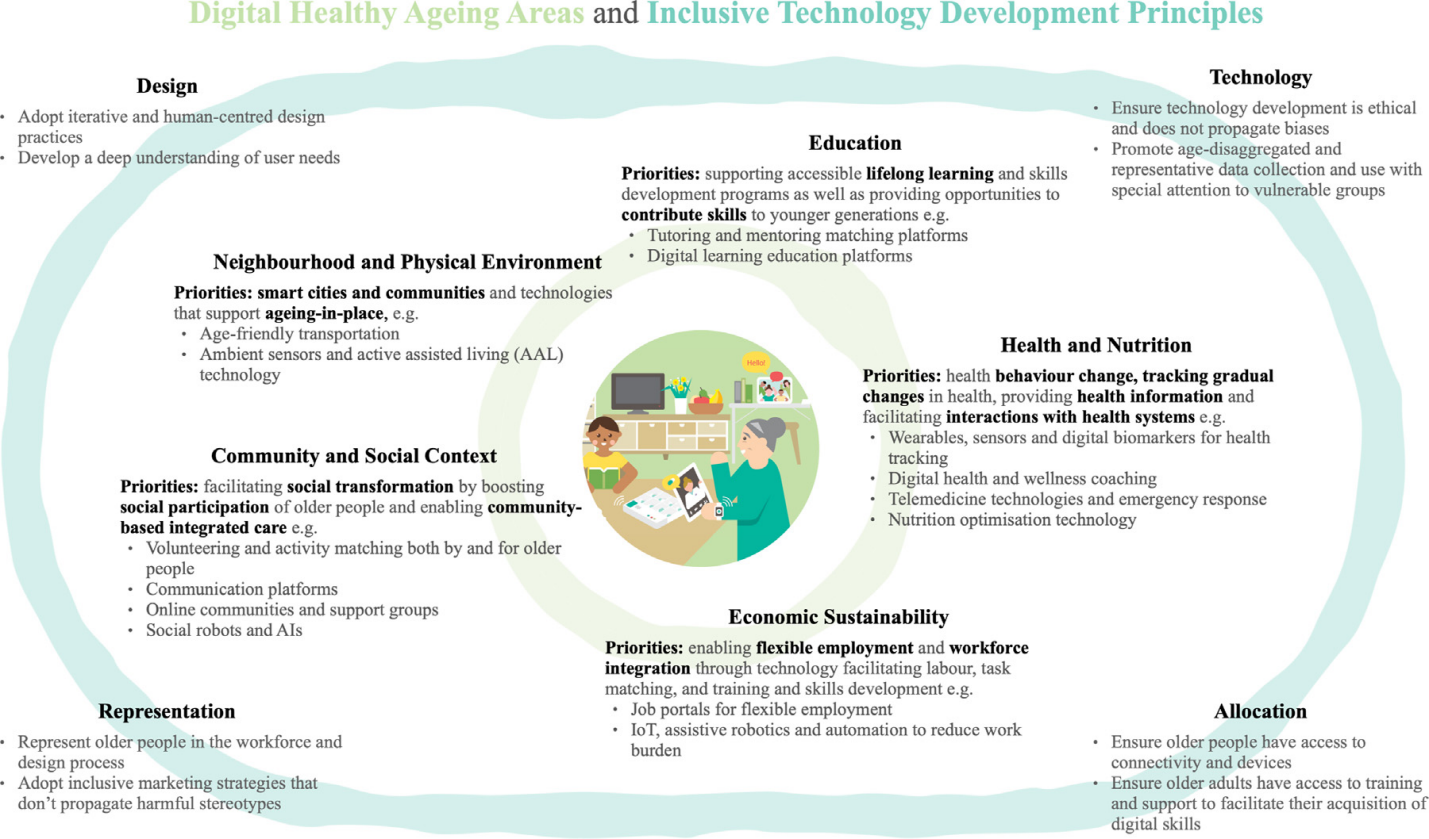
**Vision for digital healthy ageing**. Technology supports healthy ageing and maintaining functional ability by facilitating the social participation and economic sustainability of older people as well as their health. The framework provides a starting point for mapping priority areas and examples building on the social determinants of health approach to digital access by Sieck et al. (2021).^13^ Inclusive technology development guiding principles build on Chu et al. (2021)^14^ and Whittlestone et al.’s (2019)^15^ model. Economic sustainability areas are inspired by the Asian Economic Integration Report 2019/2020.^16^ The central image was adapted from WHO (2021).^17^

More holistic approaches and an understanding of technology as a tool for delivering value as opposed to the core value proposition are key to achieving digital healthy ageing. Based on this belief, we propose a vision for an integrated approach to digital healthy ageing (Fig. 2) that widens the focus of digital health for older people beyond the traditional domains of health (such as consumer health and lifestyle applications) and health systems (such as telemedicine), to include the social determinants of health: education, neighbourhood and physical environment, economic sustainability, food, and community and social context.^13^ This framework provides an initial starting points for mapping out high-impact areas for digitally-enabled service design that can support healthy ageing. Beyond priority areas, we provide four dimensions of guiding principles for the responsible and inclusive design of technologies for older people inspired by Chu and colleagues (2021).^14^ It is important to note that the social determinants of health also interact with each other. For example, lifelong learning may occur in community-based contexts, and physical environments could be used to promote healthy behaviours through the integration of technology and designs incorporating nudges.

### Challenges and policy priorities

Policymakers have a vital role to play in promoting technological innovation supporting older people's health. Several governments are already applying technology to promote health among older people such as the S-Health mobile application in Viet Nam^18^ and the Ageing 5G mobile application in Thailand.^19^ Key policy focus areas moving forward should address the challenges of bridging the digital divide, mitigating ageism and promoting the visibility of older people through research and data.

Several studies have advocated for digital access to be considered a social determinant of health, with digital tools having an amplifying effect on other social determinants of health.^13^^,^^20^ For example, employment opportunities, educational attainment and social support systems are increasingly reliant on access to technology, as highlighted by the COVID-19 pandemic.^20^ Multi-sectorial efforts should focus on expanding access to the internet and devices, as well as investing in digital literacy, inclusive design, and training and social support structures for older people. The *Seniors Go Digital* programme in Singapore is an inspirational example of bridging the digital divide through a network of community hubs with mentors and online resources that support learning digital skills. A complementary scheme was rolled out to further provide free devices and more accessible data plans for low-income seniors.^21^ While promoting the inclusion of older people in the digital world, robust offline alternatives for platforms and processes must be maintained to prevent the marginalization of the most vulnerable individuals. Bridging the digital divide will be essential to securing a return on investment in inclusive technology for older people through improved health outcomes and social participation.

Policies should also ensure that ageism does not affect the design of technology and, on the other hand, that technologies themselves do not propagate ageism. Chu and colleagues (2021)^14^ adapt Whittlestone et al.’s (2019)^15^ framework, which dissects how cycles of injustices can be propagated through technology, to the concept of *digital ageism*. This framework identifies four mutually reinforcing elements that perpetuate digital ageism: representation (e.g. stereotypes of older people as being digitally illiterate and invisibility of older people's aspirations), design (e.g. underrepresentation of older people from the design process), technology (e.g. missingness of data representative of healthy older adults) and allocation (e.g. limited internet access and technology predominantly focused on healthcare).^14^ The varied ways in which digital technologies, including AI, pose a risk to marginalized groups, for example by perpetuating biases, have been widely addressed in the context of racism and sexism but are often neglected in the context of age discrimination.^14^ Policy-makers have a vital role to play in mitigating ageism and promoting more diverse representations of older adults more broadly through multi-sectorial efforts. Furthermore, forecasting, assessing and mitigating the risks of technologies, including AI, for older people will be crucial to addressing these serious risks through proactive regulation and policies.

Finally, the public sector can drive change in the inclusive development of technologies for older people by promoting research on older adults and bridging the so-called data gap.^3^ In several countries, both coverage and granularity of data on older adults are inadequate, as exemplified by national statistics frequently categorizing “65+” as a single demographic stratum. Governments should adopt and promote practices of better disaggregation of data concerning older people and enhance the representation of particularly vulnerable groups among them, such as the very old, people with dementia, and groups in which intersectionality results in unique perspectives, such as ethnic minorities and women.^3^ The visibility of older people in data and surveillance systems will be essential not only for improving policies and programmes, but also for developing equitable digital tools relying on such data. It is vital for policymakers to advocate for participatory design and to collaborate with the private sector and academia. While strong policies are key to achieving digital healthy ageing, public sector efforts alone are not sufficient and must be complemented through strong partnerships with the private sector.

### Opportunities for private sector stakeholders and academia

The so-called “silver economy” constitutes a significant opportunity for the private sector. World Data Lab estimates that the spending power of people above the age of 60 accounts for 15% of the total spending power in Asia, and 28% of that in Europe. By 2030, the total spending power of this demographic in Asia is expected to increase by 103%.^22^ Private sector players will benefit from involving older people in the design of products to better serve this demographic with digital tools. This can be achieved both by supporting the representation of older adults among designers (e.g. by implementing flexible employment opportunities) and through the application of human-centred design methodologies.

Living Lab methodologies are particularly promising human-centred approaches that have been successfully applied for a variety of design goals.^23^, ^24^, ^25^ Ballon et al. (2005) define Living Labs as “experimentation environment[s] in which technology is given shape in real-life contexts and in which (end) users are considered ‘co-producers’”.^26^ The application of such user-driven practices and their institutionalization through dedicated spaces and communities can catalyse innovation for older adults. While powerful, living lab approaches may not be appropriate for all product application domains and across all stages of design. For example, their use may not accommodate ideas at very early stages and might be limiting when exploring perceptions and attitudes regarding future-oriented visions that may not be readily demonstrated. Academia can play an important role in documenting, formalizing and standardizing these methodologies. For example, the Vitalise Harmonization Body (https://vitalise-project.eu/harmonisation-body/) is an international effort that aims to create a common framework for understanding and rolling out Living Lab methodologies while promoting open innovation and research. Additional efforts should be directed towards a better segmentation of older people as consumers and towards better understanding of their diverse needs and behaviours, particularly in underserved contexts including low-resource settings.

Existing literature can provide a starting point for better understanding older people as users in the context of product development. Lee and Coughlin (2015) produced a synthesis of ten factors that act as facilitators or determinants of older people's use of technology.^4^ This set of factors provides a more holistic framework beyond just the physical aspects of products, encompassing individual characteristics, social contexts, delivery channels and technology characteristics. These are value, usability, affordability, accessibility, technical support, social support, independence, experience, emotion and confidence. In the context of independence, for example, authors highlight how older people can be reluctant to adopt technologies that signal dependency and frailty, such as personal emergency alarms worn as pendants, when the same functions can be embedded more subtly, for example in watches.^4^ This demonstrates how social and psychological factors should not be overshadowed by functionality in the design of technologies for older people.

Finally, it is important to note that all private sector players, and not just those actively targeting the silver market, should engage in more inclusive design and marketing strategies. Nielsen's 2014 Global Survey about Ageing, which polled over 30,000 internet respondents in 60 countries on consumer needs related to ageing, found that 51% of respondents thought that advertisements did not reflect older people's needs. Feedback on product design included widespread design issues such as difficult-to-read product labels and difficult-to-open packaging.^27^^,^^28^ These findings demonstrate that a cross-cutting transformation across all products and services is required to achieve a more inclusive and age-friendly society.

## Conclusion

In conclusion, the engagement of older people in the design process through the application of lean start-up and human-centred design approaches is key to the development of acceptable digital health innovations for older people. Engaging older adults early on in the prototyping of Agatha provided a deeper understanding of older people's conception of healthy ageing, ultimately resulting in the redesigning of Agatha as a coach that more proactively educates older people with the goal of empowering them to adopt healthy behaviours. This iterative process yielded significant improvements in user feedback. Proactively engaging users also uncovered the need to promote more holistic approaches to healthy ageing that should translate to integrated digital tools that support behaviours that directly or indirectly impact health, as opposed to driving a disease-oriented approach to health.

Building on the experience of prototyping Agatha, we provide our vision for moving beyond digital health for older people and towards achieving digital healthy ageing. We sketch priority areas for technological development, based on a social determinants of health approach, and synthesize inclusive design principles. This is in line with the Regional Action Plan for Healthy Ageing in the Western Pacific,^3^ which calls for a societal and health systems transformation, the integration of health and non-health services, a lifelong approach to health, the provision of community-based integrated care, the application of social and technological innovations, and the strengthening of data and research on older people to achieve a healthy ageing society. Policymakers can build towards the digital healthy ageing vision by focusing on bridging the digital divide, mitigating ageism and ensuring the visibility of older people in data and research. The private sector can benefit from the silver market opportunity by driving inclusive design rooted in a better understanding of the diverse needs, interests and aspirations of older people.

## Contributors

MIG drafted the article with significant inputs from the rest of the team. AL and GG ideated and led the development of Agatha with support from MC, MIG and YW. YW coordinated and managed all research projects and update requests related to Agatha. SX led the engineering team developing the prototype of Agatha.

## Declaration of interests

We declare no competing interests.
